# A NOTCH feed-forward loop drives reprogramming from adrenergic to mesenchymal state in neuroblastoma

**DOI:** 10.1038/s41467-019-09470-w

**Published:** 2019-04-04

**Authors:** Tim van Groningen, Nurdan Akogul, Ellen M. Westerhout, Alvin Chan, Nancy E. Hasselt, Danny A. Zwijnenburg, Marloes Broekmans, Peter Stroeken, Franciska Haneveld, Gerrit K. J. Hooijer, C. Dilara Savci-Heijink, Arjan Lakeman, Richard Volckmann, Peter van Sluis, Linda J. Valentijn, Jan Koster, Rogier Versteeg, Johan van Nes

**Affiliations:** 1 Department of Oncogenomics, Amsterdam UMC University of Amsterdam, Meibergdreef 9, 1105 AZ Amsterdam, The Netherlands; 2 Department of Pathology, Amsterdam UMC University of Amsterdam, Meibergdreef 9, 1105 AZ Amsterdam, The Netherlands

## Abstract

Transition between differentiation states in development occurs swift but the mechanisms leading to epigenetic and transcriptional reprogramming are poorly understood. The pediatric cancer neuroblastoma includes adrenergic (ADRN) and mesenchymal (MES) tumor cell types, which differ in phenotype, super-enhancers (SEs) and core regulatory circuitries. These cell types can spontaneously interconvert, but the mechanism remains largely unknown. Here, we unravel how a NOTCH3 intracellular domain reprogrammed the ADRN transcriptional landscape towards a MES state. A transcriptional feed-forward circuitry of NOTCH-family transcription factors amplifies the NOTCH signaling levels, explaining the swift transition between two semi-stable cellular states. This transition induces genome-wide remodeling of the H3K27ac landscape and a switch from ADRN SEs to MES SEs. Once established, the NOTCH feed-forward loop maintains the induced MES state. In vivo reprogramming of ADRN cells shows that MES and ADRN cells are equally oncogenic. Our results elucidate a swift transdifferentiation between two semi-stable epigenetic cellular states.

## Abstract (web summary)

Neuroblastoma includes adrenergic and mesenchymal cell types that can interconvert. Here, the authors show that this transdifferentiation is driven by a NOTCH feedforward loop that allows a swift transition between two semi-stable cellular states.

## Introduction

Development of the human embryo requires a multitude of lineage differentiation steps to generate a variety of specialized cell types from pluripotent stem cells. Experimental models of induced Pluripotent Stem Cells (iPSCs) or direct conversion of lineage-committed cells have provided a wealth of information on signaling molecules, gene transcription, and chromatin states that underlie the reprogramming of cellular fate. Lineage transdifferentiation is also observed in malignant cells.

An increasing number of human cancers appears to consist of phenotypically divergent tumor cell types, which recapitulate stages of normal development. We and others recently showed that neuroblastoma is composed of two cell types that reflect developmental stages of the adrenergic lineage^1,2^. Mesenchymal-type (MES) neuroblastoma cells resemble neural crest derived precursor cells, while adrenergic-type (ADRN) cells are committed to the adrenergic lineage. Both cell types can spontaneously interconvert, providing neuroblastoma with a high transcriptional plasticity^1^. Chemotherapy might select for the MES type cells, as suggested by enrichment of these cells in post-treatment samples and in relapses^1^. Also glioblastoma, melanoma, and oligodendroglioma include heterogeneous populations of tumor cells^3–5^. Both in glioblastoma and neuroblastoma, the more undifferentiated cell types have mesenchymal phenotypes and are more drug resistant, supporting the concept that lineage fate decisions are important drivers of therapy resistance in cancer.

The distinct cell populations detected in glioblastoma and neuroblastoma have unique enhancer and super-enhancer (SE) landscapes^1,2,6^. These SEs are associated with expression of lineage transcription factors (TFs) that constitute the Core Regulatory Circuitry (CRC) for each cell type. This core set of SE associated TFs were postulated to impose lineage identity^7–9^. These TFs bind to their own SE and to SEs of the other CRC TFs. This creates a strong feed-forward loop driving high levels of CRC TF expression and thereby impose lineage identity. In neuroblastoma, we identified a MES CRC of 20 TFs and an ADRN CRC of 18 TFs^1^. Several ADRN TFs are indeed proven to bind each other’s SEs^1,2^. Overexpression of PRRX1, a MES-specific CRC TF, was found to reprogram the transcriptional- and epigenetic landscapes of ADRN cells towards a MES state^1^. This shows that CRC TFs are potent inducers of lineage identity.

The CRC of MES cells included *NOTCH2* and *MAML2* that are transcriptional activators of the NOTCH pathway. The NOTCH signaling cascade is an evolutionary conserved cell to cell signaling pathway that imposes cell identity switches during development^10,11^ and can induce a motile phenotype in neuroblastoma cells^12^. Ligands of the Delta-like (Dll) or Jagged families activate full-length NOTCH receptors on neighboring cells^11^, resulting in proteolytic cleavage of NOTCH and generation of an intracellular NOTCH-IC domain^13^. NOTCH-IC translocates to the nucleus, where it forms a transcriptional complex with a mastermind-like (MAML) co-activator and the DNA-binding protein CSL. This complex regulates expression of Notch target genes^14–16^ including lineage-specific TFs to instruct cell fate decisions^10^.

Here, we investigate how a robust cancer cell type can undergo a fast and nearly complete transdifferentiation to an alternative cellular state. Expression of an inducible NOTCH-IC transgene activates an endogenous feed-forward loop among NOTCH receptors and results in transcriptional and epigenetic reprogramming of ADRN cells to a MES state. Our findings reveal how a semi-stable cancer cell type is susceptible to reprogramming by a feed-forward cascade of core lineage TFs.

## Results

### The CRC of MES cells includes NOTCH pathway genes

**Fig. 1:**
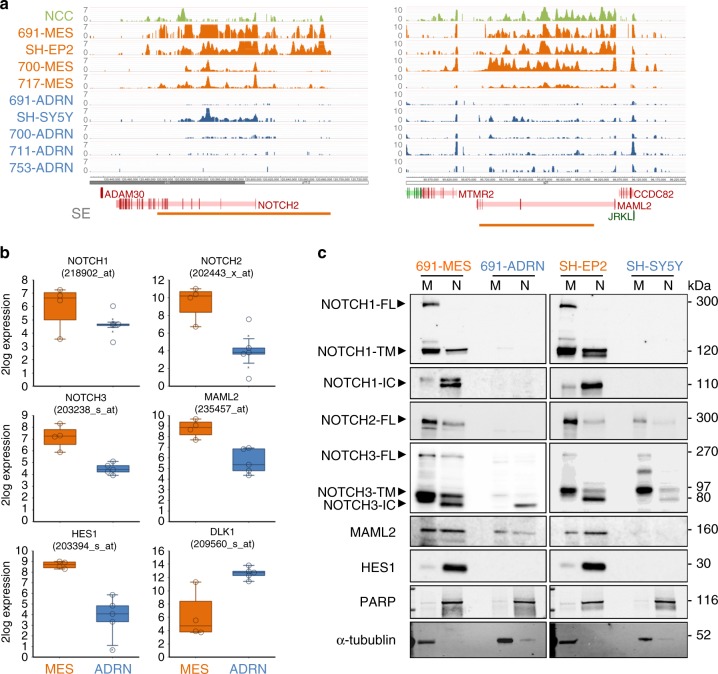
The core regulatory circuitry of MES cells includes transcription and activation of the NOTCH pathway. **a** ChIP-sequencing profiles of H3K27ac mark in neural crest cells (NCC, in green), MES neuroblastoma cells (691-MES, SH-EP2, 700-MES, 717-MES, in orange) or ADRN neuroblastoma cells (691-ADRN, SH-SY5Y, 700-ADRN, 711-ADRN, 753-ADRN, in blue). The number of reads per 20 million mapped reads is shown on the *y*-axis. Chromosomal gene position is shown on the *x*-axis. Super-enhancers (SE) are indicated by horizontal bars. Gene color depicts orientation on sense (green) and anti-sense (red) DNA strands. **b** Box plots of mRNA expression for the four MES cell lines and five ADRN cell lines shown in panel **a**. Whiskers denote the interval within 1.5 times the interquartile range (box edges) of the median (center line). Expression values (2log-transformed) for each cell line are indicated by open circles. Affymetrix mRNA probesets are indicated for each gene. **c** Western blot analysis of NOTCH1, NOTCH2, NOTCH3, the NOTCH co-factor MAML2 and the NOTCH target gene HES1 in membrane (M) or nuclear (N) lysates. Protein lysates were from MES- and ADRN cell line pairs of isogenic origin, depicted in orange and blue, respectively. IC intracellular domain, FL full-length, TM transmembrane. PARP and α-tubulin were used as loading controls for the nuclear- and membrane fractions, respectively. Source data are provided as a Source Data file

The MES CRC consists of 20 TFs, including *NOTCH2* and *MAML2*^1^. ChIP-sequencing analysis of H3K27ac revealed SEs^8,9^ within the gene bodies of *NOTCH2* and *MAML2* that were specific for MES-type neuroblastoma cells (Fig. 1a). The same super-enhancers of *NOTCH2* and *MAML2* were observed in neural crest cells, corroborating the idea that MES-type neuroblastoma cells are neural crest-like^1,2^ (Fig. 1a). The SEs were associated with strong transcription of *NOTCH2* and *MAML2* mRNA. In addition, we observed MES-specific expression of *NOTCH1* and *NOTCH3* receptors as well as the NOTCH target gene *HES1* (Fig. 1b). *NOTCH1*, *NOTCH3*, and *HES1* lack a MES-specific SE. The non-canonical inhibitory ligand *DLK1*^17,18^ was associated with an ADRN SE and showed ADRN-specific expression (Fig. 1b and see^1,19^). Neither of the NOTCH ligands *JAG1*, *JAG2*, *DLL1*, *DLL3* and *DLL4* were associated with a cell-type specific SE. There was no MES- or ADRN-specific expression pattern of these ligands. We analyzed isogenic MES and ADRN cell line pairs that were derived from the same patient^1^ for NOTCH signaling activity. Activation of NOTCH receptors requires sequential proteolytic cleavages^20^. Immunoblot analysis of membrane- and nuclear fractions from MES and ADRN cell lines revealed full-length and transmembrane NOTCH proteins in the membrane fraction- and NOTCH-IC proteins in the nucleus of MES cells (Fig. 1c), indicating ligand-induced activation of NOTCH. In addition, MES cells had higher protein levels of NOTCH2, MAML2, and HES1 (Fig. 1c). We conclude that MES-specific SEs are associated with *NOTCH2* and *MAML2* and that mesenchymal neuroblastoma cells have transcriptional activation and signaling of the NOTCH pathway.

### NOTCH induces mesenchymal reprogramming of ADRN cells

**Fig. 2:**
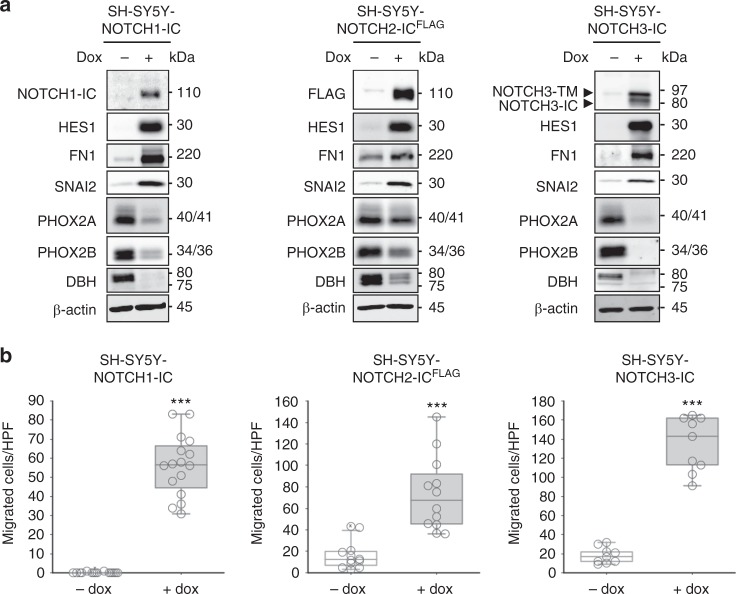
NOTCH paralogues induce a mesenchymal phenotype in ADRN neuroblastoma cells. **a** Western blot analysis of cell lysates of SH-SY5Y with inducible overexpression of NOTCH1-IC, NOTCH2-IC^FLAG^ or NOTCH3-IC. Immunoblots show NOTCH1-IC, NOTCH2-IC^FLAG^ or NOTCH3 proteins as well as protein of the known NOTCH target gene HES1 to confirm induction of each transgene. Lysates are analyzed for MES-markers (FN1 and SNAI2) and ADRN-markers (PHOX2A, PHOX2B, and DBH). β-actin was used as loading control. IC intracellular domain, TM transmembrane domain, dox doxycycline. Source data are provided as a Source Data file. **b** Transwell migration assay of SH-SY5Y cells with inducible overexpression of NOTCH1-IC, NOTCH2-IC^FLAG^ or NOTCH3-IC. Cells were allowed to migrate for 48 h. dox, doxycycline. Box plots show the number of migrated cells per High-Power Field (HPF). Whiskers denote the interval within 1.5 times the interquartile range (box edges) of the median (center line). Two-sided Student’s *t*-test assuming equal variance was used to calculate statistical difference, ***p < 0.001. Source data are provided as a Source Data file

Core Regulatory Circuitries of lineage TFs are proposed as master regulators of cell fate decisions in development and cancer^7,8^. The inclusion of NOTCH in the MES CRC urged us to test the reprogramming potential of NOTCH paralogues in the transdifferentiation of ADRN to MES lineage identity. ADRN-type SH-SY5Y cells were equipped with inducible expression of either NOTCH1-IC, NOTCH2-IC^FLAG^ or NOTCH3-IC paralogs (Fig. 2a, see Methods). All NOTCH-IC transgenes induced expression of *HES1*, confirming activation of the NOTCH pathway. Expression of each NOTCH paralog induced the MES marker proteins FN1 and SNAI2, while protein levels of ADRN markers PHOX2A, PHOX2B, and DBH were decreased (Fig. 2a). Since MES-type neuroblastoma cells are highly migratory^1^, we assessed cell motility and observed that each NOTCH-IC paralog increased migration in transwell migration assays (Fig. 2b). Thus, each of the NOTCH1-IC, NOTCH2-IC, and NOTCH3-IC paralogs induces a MES-like transition of ADRN cells.

**Fig. 3:**
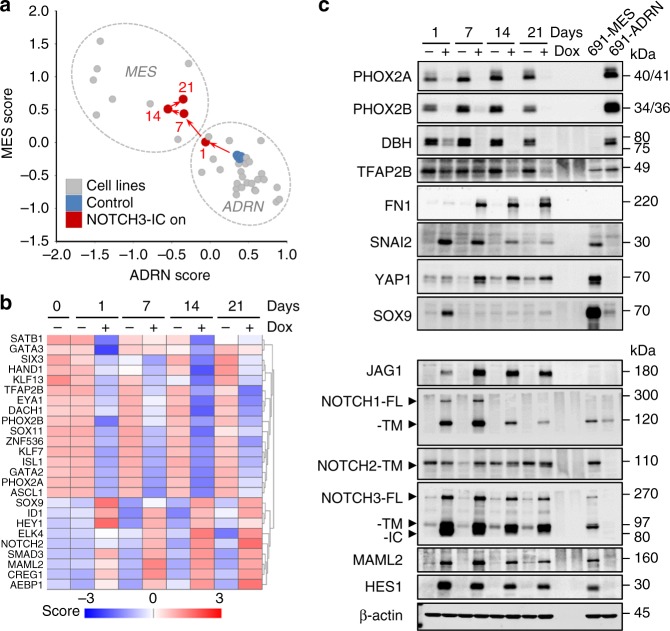
NOTCH3-IC is a master regulator of ADRN-to-MES reprogramming **a**. Analysis of mRNA signature scores for MES and ADRN cells in a panel of neuroblastoma cell lines and a time series of NOTCH3-IC induction. Control (-dox) SH-SY5Y-NOTCH3-IC cells are depicted in blue; SH-SY5Y cells with induction of NOTCH3-IC (+dox) are shown in red. Time of NOTCH3-IC induction is indicated in days. Dashed circles group cell lines of MES- or ADRN-type as previously determined^1^. **b** Z-score mRNA expression of core super-enhancer associated transcription factors specific for ADRN or MES cells. Time of NOTCH3-IC induction (in days) is indicated above. dox, doxycycline. **c** Western blot analysis showing proteins of ADRN-markers (PHOX2A, PHOX2B, DBH, and TFAP2B), MES-markers (FN1, SNAI2, YAP1, and SOX9) and members of the NOTCH pathway (JAG1, NOTCH1, NOTCH2, NOTCH3, MAML2, and HES1) in SH-SY5Y with inducible overexpression of NOTCH3-IC. Time of NOTCH3-IC induction is indicated above the western blots. Protein lysates from 691-MES and 691-ADRN cells are loaded on the same blot as a reference for MES and ADRN states, respectively. β-actin was used as loading control. dox, doxycycline. Source data are provided as a Source Data file

We selected NOTCH3-IC for further in-depth analysis as it showed the strongest repression of ADRN lineage markers (Fig. 2a). In two additional ADRN neuroblastoma cell lines, we confirmed that NOTCH3-IC induced a MES marker profile and a migratory phenotype (Supplementary Fig. 1a–c). To test the kinetics of NOTCH3-IC induced mesenchymal reprogramming, we performed a time course gene expression analysis of up to 21 days of NOTCH3-IC induction. RNA was harvested at time points of 1, 7, 14, and 21 days of NOTCH3-IC induction and analyzed by Affymetrix gene expression profiling. We calculated scores for MES and ADRN signatures (see ref. ^1^ and Methods for calculation of MES and ADRN signature scores) to follow the cell fate of SH-SY5Y cells with NOTCH3-IC expression. After 1 day of NOTCH3-IC expression cells had markedly reduced the ADRN score, but still grouped with a panel of ADRN cell lines (Fig. 3a). After 7 days the cells had achieved a strong MES score, maintained a reduced ADRN score and grouped with a panel of MES-type cells. Thus ADRN-type SH-SY5Y cells undergo a step-wise transition to an induced MES state after 7 days of NOTCH3-IC expression (Fig. 3a and Supplementary Data 1). Principal Component Analysis of these cell lines using all expressed genes instead of MES and ADRN signatures, showed that principal components 1 and 2 resolved the pattern of MES and ADRN cells and confirmed that NOTCH3-IC induced reprogramming to a MES state (Supplementary Fig. 2). Analysis of the Core Regulatory Circuitries of TFs (see ref. ^1^) revealed that 16 out of 18 ADRN core TFs had strongly reduced expression, while 8 out of 20 MES core TFs were induced after three weeks of NOTCH3-IC induction (Fig. 3b). The patterns of regulation showed strong differential expression of most ADRN TFs at *T* = 24 h and gradual regulation of MES TFs at later time points (Fig. 3b) that were confirmed for a selected set of marker proteins (Fig. 3c). These results indicate a nearly complete repression of the ADRN CRC, but induction of the MES CRC is partial. MES reprogramming of ADRN cells induced chemo-resistance (Supplementary Fig. 3), consistent with our previous finding that MES cells are chemo-resistant as compared to ADRN cells^1^. We conclude that NOTCH3-IC is on top of a hierarchy regulating core transcriptional modules of lineage differentiation and suggest that repression of the ADRN lineage is an essential, early phase during mesenchymal reprogramming.

### Remodeling of lineage-associated SEs

**Fig. 4:**
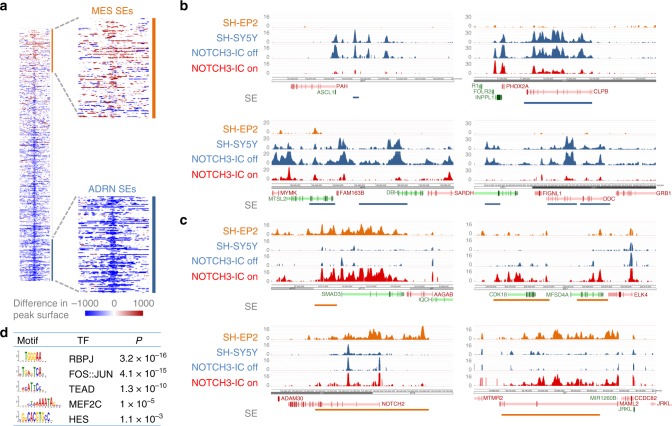
NOTCH3-IC induces epigenetic remodeling of super-enhancer landscapes. **a** Analysis of H3K27ac marked super-enhancer regions (*n* = 1662) that were previously shown to be associated with MES or ADRN cells in neuroblastoma^1^. Shown are changes of H3K27ac signal in SH-SY5Y cells with induced expression of NOTCH3-IC (*T* = 7 days of induction). Red (increased) or blue (decreased) H3K27ac signal of the super-enhancers is depicted by horizontal bars on the genomic regions. Expanded views show the super-enhancer (SE) regions that distinguish MES and ADRN lines (indicated by orange and blue vertical bars, respectively) (right). **b**, **c** H3K27ac profiles in SH-EP2, SH-SY5Y and in SH-SY5Y cells with (NOTCH3-IC on) or without (NOTCH3-IC off) 7 days of doxycycline-induced expression of NOTCH3-IC. Shown are examples of **b** ADRN-specific SE-associated TF genes (*ASCL1*, *PHOX2A*) and ADRN lineage genes (*DBH*, *DDC*). In **c**, examples of MES-specific SE-associated TF genes (*SMAD3*, *ELK4*, *NOTCH2*, *MAML2*) are shown. SEs associated with ADRN- and MES cells are indicated by blue and orange horizontal bars, respectively. Gene color depicts orientation on sense (green) and anti-sense (red) DNA strands. **d**. Motif analysis on regions with increased H3K27ac signal. Statistical significance of enrichment was calculated using Fisher’s exact test with Bonferroni correction

ADRN and MES neuroblastoma cells have unique SE landscapes that are associated with lineage identity and expression of CRC TFs^1^. We tested the function of NOTCH3-IC in remodeling of the SE landscape during reprogramming of ADRN cells to an induced MES state. SEs were identified by ChIP-sequencing for H3K27ac^8,9^ on SH-SY5Y cells after seven days of NOTCH3-IC induction. Analysis of 1662 SEs previously shown to distinguish MES from ADRN cells^1^, revealed strong reductions of H3K27ac signal at many ADRN SEs and mild induction of MES SEs (Fig. 4a). The SEs of core ADRN lineage TFs (e.g. *PHOX2A*, *ASCL1*) showed a severely diminished H3K27ac signal (Fig. 4b). In addition, the SEs of ADRN lineage marker genes, such as *DBH* and *DDC*, showed extensive reduction of H3K27ac (Fig. 4b), confirming suppression of the ADRN state at lineage-specific enhancers. Conversely, SEs that associated with MES core TFs (e.g. *SMAD3*, *ELK4*) were induced by NOTCH3-IC (Fig. 4c).

The SH-SY5Y cell line emanated from the SK-N-SH neuroblastoma cell line, from which the SH-EP2 cell line is derived as well^21^. SH-EP2 has a mesenchymal phenotype, providing us with a reference for the SE switches induced by NOTCH in SH-SY5Y. Figure 4 shows examples of H3K27ac signal at MES-specific SE regions that were induced by NOTCH3-IC and were remarkably similar to endogenous H3K27ac patterns in SH-EP2. Interestingly, repression of ADRN SEs was relatively strong as compared to induction of MES SEs (Fig. 4a), suggesting that repression of ADRN lineage genes is required for mesenchymal reprogramming. To analyze a putative direct role for NOTCH in reprogramming the enhancer landscape, we searched for TF binding motifs on regions where reprogramming had increased H3K27ac signal (see Methods). Regions with increased H3K27ac were enriched for *RBPJ* and *HES* motifs (Fig. 4d and Supplementary Data 2), suggesting a direct regulatory role for NOTCH in enhancer remodeling. These data thus identify NOTCH3-IC as a TF that can induce remodeling of the epigenetic landscape and reprogram ADRN cells to a MES lineage.

### NOTCH induces a feed-forward cascade from endogenous loci

**Fig. 5:**
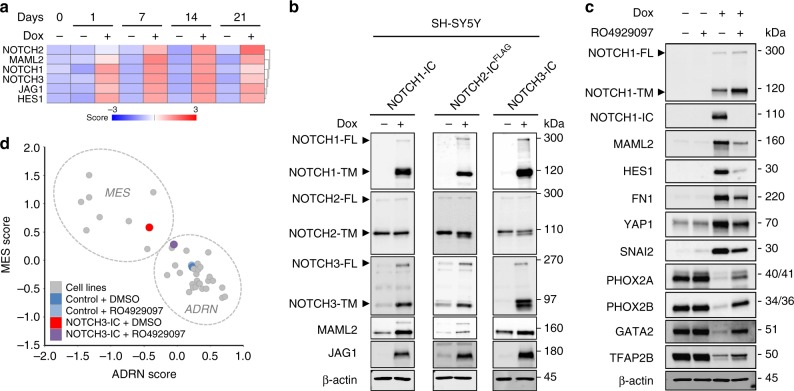
NOTCH-IC paralogues induce a reciprocal feed-forward signaling cascade. **a** SH-SY5Y cells with inducible overexpression of NOTCH3-IC were profiled for gene expression up to 21 days. Z-scores of mRNA expression values are shown for *JAG1*, *NOTCH1*, *NOTCH2*, *NOTCH3*, *MAML2*, and *HES1*. The transcription of NOTCH receptors from endogenous loci is measured using probesets located in the 3′UTRs of *NOTCH1* (probeset 218902_at), *NOTCH2* (probeset 202443_x_at) and *NOTCH3* (probeset 203238_s_at). Notably, the 3′UTR is absent in the NOTCH3-IC transgene. **b**. Western blot analysis of NOTCH1, NOTCH2, NOTCH3, MAML2, and JAGGED1 proteins in SH-SY5Y with NOTCH1-IC, NOTCH2-IC^FLAG^ or NOTCH3-IC inducible overexpression. β-actin was used as loading control. Time of induction was 96 h. Source data are provided as a Source Data file. **c** Western blot analysis of SH-SY5Y cells with (+dox) or without (−dox) NOTCH3-IC expression that were cultured in the presence (+) or the absence (−) of the gamma-secretase inhibitor RO4929097. Protein lysates were analyzed for NOTCH pathway genes (NOTCH1, MAML2, HES1), MES markers (FN1, YAP1, SNAI2) and ADRN markers (PHOX2A, PHOX2B, GATA2, TFAP2B). β-actin was used as loading control. dox, doxycycline. Source data are provided as a Source Data file. **d** Analysis of MES and ADRN mRNA signature scores in SH-SY5Y cells with NOTCH3-IC induction that were treated with the gamma-secretase inhibitor RO4929097. Control cells (-dox) treated with DMSO or RO4929097 are shown in dark- and light blue, respectively. NOTCH3-IC expressing cells with DMSO or RO4929097 are shown in red and purple, respectively. Cells were induced with doxycycline and treated with RO4929097 for 14 days. Dashed circles indicate groups of MES- or ADRN-type cell lines as previously determined^1^

The surprising fast MES transition of ADRN SH-SY5Y cells urged analysis of the changes in gene expression in the early phase of this process. The mRNA expression of the endogenous full-length *NOTCH3* as well as *NOTCH1* and *NOTCH2* receptors appeared to be induced by the NOTCH3-IC transgene within 24 h (Fig. 5a). In addition, NOTCH3-IC induced transcription of the NOTCH-ligand *JAG1*, the co-factor *MAML2* and the NOTCH target gene *HES1* (Fig. 5a). We tested whether the induced NOTCH receptors were activated. Western blot analysis confirmed induction of full-length NOTCH1 and NOTCH3 proteins by NOTCH3-IC (Fig. 3c). Ligand-induced activation of full-length NOTCH receptors results in transmembrane- and intra-cellular domain proteins of lower molecular weight. We observed increased levels of transmembrane protein epitopes of NOTCH1 and NOTCH3 receptors as well as NOTCH2-IC proteins, indicating that NOTCH receptors were activated by ligands and processed to signaling proteins (Fig. 3c). This activation of NOTCH receptors was accompanied by an induction of JAG1, MAML2, and HES1 (Fig. 3c), the latter confirming the transcriptional activity of NOTCH3-IC. These results indicated that a NOTCH3-IC transgene can start a positive feed-forward loop in the NOTCH pathway. We examined the epigenetic mechanisms of this transcriptional feed-forward. Analysis of H3K27ac revealed that de novo SEs were established at *NOTCH2* and *MAML2* genes, while the promoter regions of *JAG1*, *NOTCH1*, *NOTCH3*, and *HES1* became marked by H3K27ac after induction of NOTCH3-IC (Fig. 4c). Thus, a single reprogramming factor induces a transcriptional feed-forward cascade of NOTCH from endogenous loci that is associated with epigenetic remodeling.

To test whether this transcriptional amplification is a general property of NOTCH paralogues, we overexpressed NOTCH1-IC, NOTCH2-IC^FLAG^ or NOTCH3-IC in SH-SY5Y cells and analyzed the endogenous transcriptional regulation of each of these NOTCH paralogues by qRT-PCR (see Methods). Each transgenic NOTCH-IC paralogue induced expression of the corresponding endogenous full-length NOTCH receptor, showing an autoregulatory feed-forward loop (Supplementary Fig. 4). Furthermore, each NOTCH-IC transgene induced transcription of the paralogous NOTCH genes from endogenous loci (Supplementary Fig. 4), starting a transcriptional feed-forward cascade among NOTCH receptor paralogues. In addition, *JAG1* and *MAML2* were induced (Supplementary Fig. 4). The full-length NOTCH receptors were processed to NOTCH transmembrane proteins of lower molecular weight, indicative of their activation (Fig. 5b). To test whether this NOTCH feed-forward cascade is required for ADRN-to-MES reprogramming, we applied the gamma-secretase inhibitor RO4929097 to SH-SY5Y cells during NOTCH3-IC induction. RO4929097 attenuated formation of NOTCH1-IC and diminished induction of HES1. Western blot analysis for a selected set of marker proteins showed that treatment with RO4929097 attenuated the induction of MES markers and prevented silencing of ADRN markers (Fig. 5c). Gene expression profiling revealed that RO4929097 prevented the ADRN-to-MES transdifferentiation induced by NOTCH3-IC (Fig. 5d). We conclude that a reciprocal feed-forward loop generates a NOTCH cascade with high signaling levels that is required for mesenchymal reprogramming.

### Transient NOTCH3-IC induces commitment to a MES lineage

**Fig. 6:**
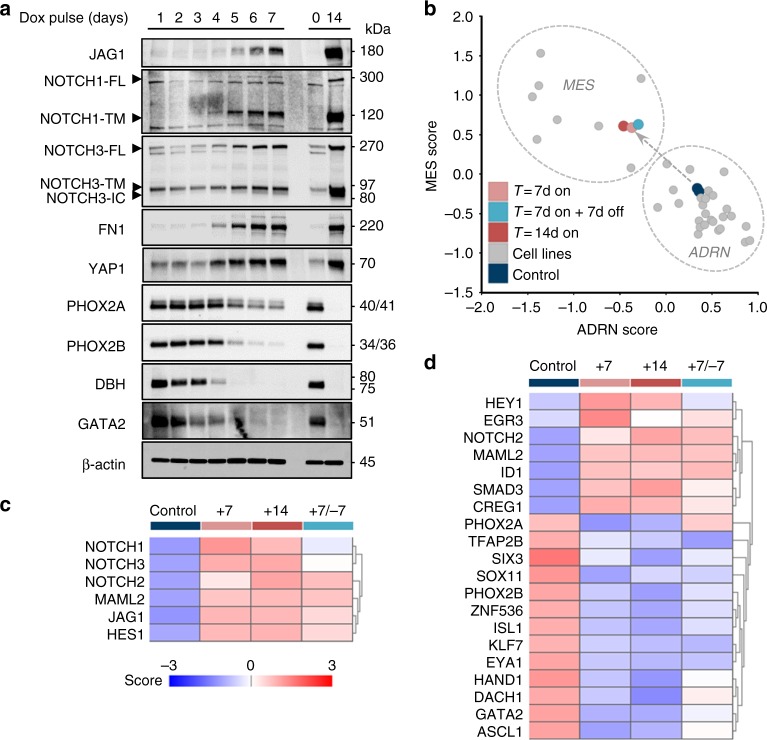
A transient phase of NOTCH3-IC expression induces commitment to a MES phenotype. **a** Western blot analysis of transient NOTCH3-IC induction. Time of doxycycline (dox) induction is indicated above the blots. Induction was followed by doxycycline wash-out and all samples were harvested after *T* = 14 days. Immunoblots show NOTCH pathway proteins (JAG1, NOTCH1, and NOTCH3), MES-markers (FN1 and YAP1) and ADRN-markers (PHOX2A, PHOX2B, DBH, and GATA2). As a positive reference, a sample of continued NOTCH3-IC induction for *T* = 14 days was run on the same gel (right). β-actin was used as loading control. Source data are provided as a Source Data file. **b** Summed z-scores of mRNA signatures for MES and ADRN genesets. Non-induced control (−dox) cells are shown in dark blue. Persistent NOTCH3-IC induction of *T* = 7 and 14 days are shown in pink and red, respectively. A transient phase of NOTCH3-IC induction (*T* = 7 days + doxycycline, followed by *T* = 7 days - doxycycline wash-out) is shown in light blue. As a reference, various neuroblastoma cell lines are shown in gray dots and grouped by dashed lines according to MES and ADRN phenotypes (see ref. ^1^). **c**, **d** mRNA analysis (z-score of expression) of C) NOTCH pathway genes and D) core transcription factors of MES and ADRN lineages. + and—indicate culture conditions with or without doxycycline, respectively. Time of doxycycline treatment is indicated in days. For color-coding of samples, see panel **c**

The generation of a strong feed-forward loop implies that a temporary activation of NOTCH signaling may trigger a full-scale transition. We therefore tested whether transient activation of the NOTCH3-IC transgene can result in a lasting activation of the feed-forward loop and mesenchymal transition. In cultures of SH-SY5Y-NOTCH3-IC, the NOTCH3-IC transgene was induced for 0 to 7 days, after which all cultures were followed until day 14 and harvested. Western blot analysis showed from 5 days of transient NOTCH3-IC expression onwards a strong induction of JAG1, full-length- and transmembrane NOTCH1 and NOTCH3, and intracellular domain of NOTCH3 (Fig. 6a). The strength of this induction increased until 7 days of transient NOTCH3-IC induction (Fig. 6a). After 5–7 days of transient NOTCH3-IC expression, the MES markers FN1 and YAP1 remained induced and the ADRN markers PHOX2A, PHOX2B, DBH, and GATA2 remained repressed until day 14 (Fig. 6a). Evidently, five days of transient NOTCH3-IC activation is sufficient to initiate and maintain the feed-forward cascade until day 14, albeit that continued NOTCH3-IC induction for 14 days shows stronger protein signals (Fig. 6a).

We then designed an experiment to analyze the extent of ADRN-to-MES transition after transient NOTCH activation. NOTCH3-IC was transiently induced for 7 days followed by 7 days without NOTCH3-IC induction (+7/−7). As controls, the NOTCH3-IC transgene was not induced (−14), or induced for 7 days (+7) or 14 days (+14). mRNA profiling showed that the +7/−7, the +7 and the +14 cells all reached a comparable ADRN-to-MES transition (Fig. 6b). NOTCH pathway genes remained up-regulated in the +7/−7 as compared to the +14 cells, albeit at slightly reduced levels as revealed by mRNA profiling and qRT-PCR (Fig. 6c and Supplementary Fig. 5). Also core MES TFs remained induced and core ADRN TFs remained repressed (Fig. 6d). We conclude that transient expression of exogenous NOTCH3-IC elicits an endogenous NOTCH feed-forward loop and is sufficient to maintain reprogramming of the induced MES state.

### ADRN-to-MES reprogramming is tumorigenic in vivo

**Fig. 7:**
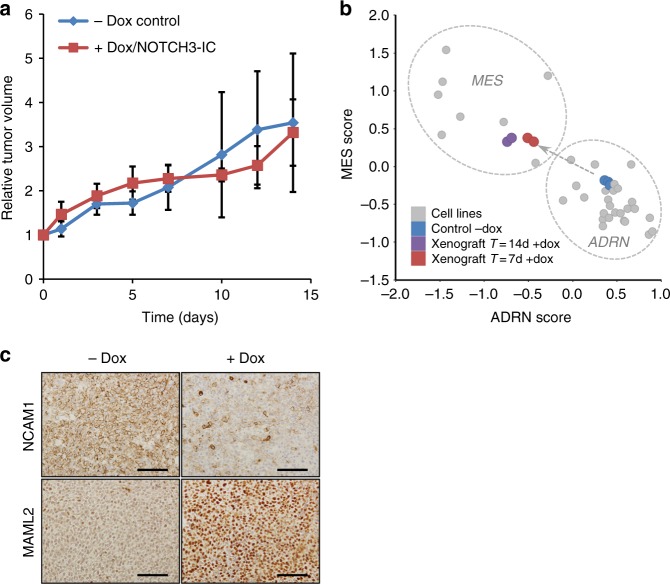
Induction of ADRN-to-MES reprogramming is tumorigenic in vivo. **a** Growth of SH-SY5Y-NOTCH3-IC xenograft with (+dox, red) or without (−dox, blue) doxycycline in the drinking water. Error bars represent standard deviation. *Y*-axis shows relative tumor growth; *x*-axis depicts time in days. Source data are provided as a Source Data file. **b** mRNA analysis of ADRN and MES signature scores of SH-SY5Y-NOTCH3-IC xenograft tumors with induction of NOTCH3-IC. Non-induced control tumors (*n* = 4) are depicted in blue, tumors with 7 or 14 days NOTCH3-IC expression (*n* = 4) are shown in red and purple, respectively. Dashed lines group various neuroblastoma cell lines (gray dots) according to MES and ADRN phenotypes for reference (see ref. ^1^). **c** Immunohistochemistry of NCAM1 and MAML2 on tumors with (+dox) or without (−dox) 7 days of NOTCH3-IC induction. dox, doxycycline. Scale bar, 100 µM

We previously found that FACS-sorted ADRN and MES cells are both oncogenic in vivo and generated heterogeneous tumors^1^. We now investigated whether neuroblastoma tumors remained oncogenic after massive trans-differentiation to the MES phenotype. SY5Y-NOTCH3-IC cells were inoculated in immunodeficient mice. When palpable tumors reached about 200 mm^3^ in size, doxycycline was added to the drinking water to induce NOTCH3-IC expression. Tumors with NOTCH3-IC induction maintained equally aggressive growth kinetics as control tumors (*n* = 3 mice per group, Fig. 7a).

We analyzed the transcriptome of ADRN SH-SY5Y tumors after 7 or 14 days of NOTCH3-IC induction in vivo. The MES and ADRN signatures^1^ were used to test an ADRN-to-MES transition. Tumors with NOTCH3-IC expression showed downregulation of the ADRN signature and induction of the MES signature comparable to the effect of NOTCH3-IC in vitro (Fig. 7b). ADRN lineage markers, such as *DBH*, *DDC*, and *TH* were repressed, while MES lineage markers like *FN1*, *SNAI2*, *YAP1*, and various NOTCH pathway genes, were induced (ANOVA, *p* < 0.001; Supplementary Fig. 6a, c). Moreover, NOTCH3-IC downregulated expression of 12 out of 18 expressed TFs from the ADRN CRC, including *PHOX2A*, *PHOX2B*, and *ASCL1* (ANOVA, *p* < 0.001; Supplementary Fig. 6b).

To follow the transition of ADRN-to-MES reprogramming in vivo, we used *NCAM1* and *MAML2* as SE-associated marker genes of ADRN and MES cells, respectively (Supplementary Fig. 6a, c and Supplementary Fig. 7a–c, Figs. 3c, 4b). Non-induced control tumors expressed NCAM1 protein, but hardly expressed MAML2 (Fig. 7c). In strong contrast, tumors with NOTCH3-IC expression were largely devoid of NCAM1 but induced MAML2, indicating a homogeneous pattern of ADRN-to-MES transition (Fig. 7c).

We determined the relation of NOTCH gene expression and the MES state in a series of 498 primary human neuroblastoma^22,23^. The MES signature was significantly correlated to expression of genes from the NOTCH feed-forward cascade that included *NOTCH1*, *NOTCH2*, *NOTCH3*, *JAG1*, *MAML2*, and *HES1* (ANOVA, *p*-values between 10^–65^ to 10^–125^ for all correlations tested, Supplementary Fig. 8a–d). We conclude that NOTCH pathway genes are co-regulated with a MES state in human neuroblastoma and that an induced mesenchymal reprogramming of neuroblastoma cells does not affect the tumorigenicity of neuroblastoma cells in vivo.

## Discussion

Neuroblastomas are pediatric tumors that originate from the developing peripheral sympathetic nervous system. They occur in the adrenal medulla and at various sites along the routes of migrating neural crest cells. During normal development, lineage-specific enhancers regulate the cell-type specific transcriptomes and mark phenotypically divergent states of cellular differentiation. These epigenetic mechanisms allow cells to differentiate via remodeling of their regulatory landscapes. Differentiation follows a unidirectional hierarchy, although in exceptional cases lineage-committed cells can revert to a stem-like state^24,25^. Lineage differentiation hierarchies of normal development show parallels in cancer heterogeneity. Neuroblastoma tumors include a majority of lineage-committed ADRN tumor cells and a minority of MES tumor cells that resemble neural-crest cells^1^. These cell types can spontaneously interconvert. However, the potential for transdifferentiation and the driving mechanisms remain largely unexplored.

Here, we identified the NOTCH pathway as a driver of a dedifferentiated MES identity. Many NOTCH receptors and co-factors are expressed in MES neuroblastoma cells. Some of these, i.e. *NOTCH2* and *MAML2*, are part of the CRC of lineage TFs in MES cells^1^. *NOTCH2* and *MAML2* are highly expressed, probably as a result of their associated SE. Together, these genes drive high levels of NOTCH signaling, supported by detection of NOTCH-IC signaling domains and expression of the NOTCH target gene *HES1*. The different NOTCH paralogues can regulate unique target genes as well as a shared set of genes^26–28^. The concurrent activation of NOTCH1, NOTCH2, and NOTCH3 paralogues may therefore exert broad control on induction and/or maintenance of the MES state. Reprogramming experiments using paralogous NOTCH-IC transgenes revealed that all three NOTCH paralogues initiated transdifferentiation, while NOTCH3-IC is the strongest inducer of reprogramming ADRN cells towards a MES state. The strong transcriptional silencing of ADRN genes may result from NOTCH3-IC-specific recruitment of protein complexes and paralogue-specific transcriptional activity of NOTCH3^26,29^. The downregulation of ADRN signature genes included CRC TFs and was accompanied by severe reduction of H3K27ac at their SEs. Newly acquired MES-specific enhancers in ADRN SH-SY5Y cells arose at positions similar to the wild-type MES cell line SH-EP2, which is the isogenic counterpart of SH-SY5Y. This suggests that the epigenetic landscape in ADRN cells retains a MES-specific pattern of accessible chromatin where enhancers can arise.

A core set of stem-cell TFs can induce pluripotent stem cells from differentiated fibroblasts, albeit at low frequency and with slow kinetics^30^. During reprogramming of neuroblastoma, we observed a swift and rather homogeneous phenotypic transition upon expression of a single NOTCH-IC transgene. This suggests that the majority of cells retained the competence for reprogramming to a dedifferentiated MES state. The potential for reprogramming may be influenced by genetic or epigenetic events that occur in specific backgrounds of tumors or cancer cell-lines. For instance, an H3K27ac *NOTCH2* enhancer in SH-SY5Y may cooperate with NOTCH3-IC in the induction and/or maintenance of the MES state. At the genetic level, chromosomal gains or losses may affect essential genes that facilitate reprogramming. Nevertheless, the transcriptional states of MES and ADRN cell lines represent a general phenomenon that can exist across different genetic backgrounds^1^. The kinetics of reprogramming revealed a step-wise transition from the ADRN to a MES state, characterized by initial repression of ADRN genes, followed by induction of MES gene expression within a week. In search for a mechanism explaining this rapid transition, we found that various NOTCH-IC paralogues induced feed-forward expression of the corresponding endogenous NOTCH gene. Moreover, each NOTCH-IC cross-activated paralogous full-length NOTCH receptors. The reciprocal feed-forward loop among NOTCH paralogs can rapidly amplify the transcription of NOTCH receptors. This may explain the co-regulated expression of paralogous NOTCH receptors in MES cells, while only *NOTCH2* is associated with a SE. The NOTCH ligand *JAG1* is a target of NOTCH3-IC in ovarian cancer and in neuroblastoma (ref. ^31^ and this work), suggesting that NOTCH feed-forward cascades exist across different tumor backgrounds. While the NOTCH cascade in ovarian cancer includes regulation of *RBPJ*, this gene is not induced by NOTCH3-IC in neuroblastoma^32^. The concurrent induction of the JAG1 ligand and the MAML2 co-factor can concomitantly activate NOTCH signaling and induce an ADRN-to-MES transition. Such feed-forward loops may represent a general mechanism in which transcriptional cascades rapidly activate a signaling pathway among a population of cells, ultimately leading to repression of lineage-specific transcriptional states and induction of an alternative reprogrammed state.

Phenotypically divergent cancer cell types may cooperate to drive tumor malignancy. Small-cell lung cancer (SCLC) includes neuro-endocrine (NE) and non-NE cells. The latter endowed NE cells with metastatic capacity, indicating functional cooperation among phenotypically distinct tumor cells^33^. In other cases, subsets of cancer cells can act as a tumor-derived niche. For instance, non-NE SCLC cells provide trophic support to stimulate NE cell proliferation^34^. Tumor-derived WNT-secreting cells induce proliferation of WNT-responsive LGR5^+^ SCLC cells^35^. Tumor progression is thus supported by an inter-dependency of phenotypically distinct tumor cells. Here we showed that NOTCH activated a feed-forward loop and a swift phenotypic transdifferentiation that may establish an ecosystem formed by phenotypically heterogeneous tumor cells.

## Methods

### Cell culture

Patient-derived neuroblastoma cell lines were derived and cultured in neural stem cell (NSC) medium as described^1,36^. Serum-cultured cell lines SH-EP2 and SH-SY5Y were maintained as described^12^. Cell line authenticity was verified using Short Tandem Repeat (STR) analysis. Cells were routinely checked for the presence of mycoplasma using MycoAlert detection kit (Lonza). The gamma-secretase inhibitor RO4929097 (Selleckchem) was added to the culture medium at a final concentration of 10 µM.

### Generation of transgenic cell lines with inducible NOTCH-IC

Polyclonal SH-SY5Y Tet repressor (TetR) cells were established by lentiviral transduction of a TetR construct (Invitrogen) and selected with 3 µg/ml blasticidin. These SH-SY5Y-TetR cell-lines were subsequently transduced with pLenti4 vectors that contain cDNAs encoding human NOTCH1-IC, NOTCH2-IC^FLAG^ or NOTCH3-IC. Stable polyclonal cell lines were established using puromycin selection (4 µg/ml). All constructs were transduced at a multiplicity of infection (MOI) of 3. Transgenes were induced by addition of doxycycline to the culture medium at a final concentration of 100 ng/ml. For constitutive overexpression in 691-ADRN and in 700T-ADRN cell lines, pLenti6-NOTCH3-IC, and pLenti6-Luciferase2 (LUC2) constructs were packaged and transduced at an MOI of 3.

### Gene expression profiling and analysis of micro-array data

mRNA expression profiles of SH-EP2, SH-SY5Y, 691-MES, 691-ADRN, 700-MES, 700-ADRN, 717-MES, and 717-ADRN are available from GEO (GSE90805^1^). Total RNA from SH-SY5Y-TetR-NOTCH3-IC cells was harvested at 1, 7, 14, and 21 days of doxycycline induction and isolated using Trizol reagent (Invitrogen). RNA quality was verified on a Bioanalyzer (Agilent). RNA was hybridized on Affymetrix HG U133A plus2.0 gene chips and scanned data were normalized using the MASS5.0 algorithm. Expression data is available from GEO (GSE116893). Bio-informatic analysis of microarray data was conducted using R2 ([http://R2.amc.nl]). RNA-sequencing analysis of 498 primary human neuroblastoma had been published previously^22,23^ and is available from GEO (GSE62564) and in R2. Expression of NOTCH receptors from endogenous loci was measured using Affymetrix probesets, located in the 3′UTRs of *NOTCH1* (probeset 218902_at), *NOTCH2* (probeset 202443_x_at) and *NOTCH3* (probeset 203238_s_at). The NOTCH3-IC transgene lacks the 3′UTR and is therefore not measured using Affymetrix probeset 203238_s_at that is located in the *NOTCH3* 3′UTR.

### MES and ADRN gene signature scores

The gene expression signatures for MES and ADRN cells were calculated as previously described^1^. In short, these gene signatures were derived from mRNA expression data of four MES and ADRN cell line pairs of isogenic origin. From each cell line pair, genes with (1) significantly different expression in at least three cell line pairs, (2) with a minimum expression difference of 100, (3) a minimum of 1 present call and (4) with a consistent pattern of regulation, were selected and merged. This identified 485 MES-specific and 369 ADRN-specific genes. Gene signatures were converted in a single value per sample using the following method: Genes were rank-ordered according to their expression level and the percentile was calculated relative to the full list. The average of the percentiles for all MES and ADRN signature genes was used as a signature score and can be compared across samples.

### qRT-PCR analysis

Analysis of the NOTCH feed-forward cascade was performed by qRT-PCR on oligo-dT generated cDNA. Oligos for qRT-PCR were required to span intron-exon boundaries, if possible. Oligos for the detection of NOTCH receptors were designed in exons that were exclusively present in the full-length NOTCH mRNA and absent from the transgenic NOTCH-IC constructs, to allow detection of transcription of NOTCH receptors form the endogenous loci. Forward (F) and reverse (R) oligo sequences were NOTCH1-F (5′-CGACGTCACCCACGAGT-3′), NOTCH1-R (5′-CTGGCAGGCATTTGGCATCA-3′), NOTCH2-F (5′-ACAGCCTGTATGTGCCCTGT-3′), NOTCH2-R (5′-ACTGTCCTGTCCATTGTGGG-3′), NOTCH3-F (5′-CCGCGTGGCTTCTTTCTACT-3′), NOTCH3-R (5′-TGTTCACGCACCTGCCCAA-3′), HES1-F (5′-ATAAACCAAAGACAGCATCTGAGC-3′), HES1-R (5′-GTTCCGGAGGTGCTTCACT-3′), MAML2-F (5′-GTGGGATAAACGGAGAGCAGCA-3′), MAML2-R (5′-TTGTTTATTTGGAGGCCACCTTGT-3′), JAG1-F (5′-CGCATCGTGCTGCCTTTCAG-3′), JAG1-R (5′-GTCACGCGGATCTGATACTCAA-3′), GAPDH-F (5′-CCCCTTCATTGACCTCAACTACA-3′), GAPDH-R (5′-TTGCTGATGATCTTGAGGCTGT-3′). *GAPDH* was used as a loading control. Each experimental condition was measured in triplicate and repeated at least twice with comparable results. Two-sided Student’s *t*-test assuming equal variance was used to calculate statistical difference.

### ChIP-sequencing analysis

ChIP-sequencing profiles for H3K27ac in MES- and ADRN cell lines are available from GEO (GSE90805) and were analyzed as described^1^. Enhancers (H3K27ac) were determined by ChIP-sequencing in SH-SY5Y-TetR-NOTCH3-IC cells after 7 days of doxycycline induction. ChIP-sequencing using H3K27ac antibody (4729, Abcam) was performed as described^1^. H3K27ac ChIP-sequencing data from SH-SY5Y-TetR-NOTCH3-IC cells are available from GEO (accession number GSE116893).

### Motif-enrichment analysis

RSEG diff (using default arguments) was used to select regions with increased H3K27ac in SY5Y-NOTCH3-IC cells treated with 7 days of doxycycline and compared to non-induced control cells. Motif analysis was performed on the extreme end (top500 or top1000 induced H3K27ac regions) of the RSEG diff list, when sorted on (col e *col f ( = directional surface)). Motif enrichment analysis (MEME suite AME ([http://meme-suite.org/tools/ame])) was performed on the H3K27ac increasing regions using the JASPAR database (JASPAR2018_CORE_vertebrates_non-redundant). Significance of enrichment was calculated using Fisher’s exact test with Bonferroni correction.

### Western blot and cell fractionation analysis

Western blotting was performed according to standard protocols. Total cell lysates were made in RIPA-buffer. Cell fractionation was performed using ProteoExtract S-PEK kit (Calbiochem, 539790) according to the manufacturer’s instructions. Protein was transferred to nitrocellulose membrane (GE Healthcare, RPN203D). Membranes were blocked for 1 h at RT, incubated at 4 °C overnight with primary antibody and incubated for 1 h at RT with secondary antibodies in either 2% PBA (GE Healthcare, RPN418), 5% ELK or OBB (LI-COR, 829–31080) in PBS 0.1% TWEEN (Sigma, P1379).

Primary antibodies NOTCH1 (4380), NOTCH1-IC (4147), NOTCH2 (4530), NOTCH3 (5276), MAML2 (6988), HES1 (11988), DBH (8586), VIM (5741), SNAI2, (9585), JAGGED1 (2620), GATA2 (4595), YAP1 (4912), SOX9 (82630), TFAP2B (2509), PARP (9542), and FLAG (2368) were obtained from Cell Signaling Technology. Other primary antibodies were FN1 (AF1918, R&D systems), PHOX2A (sc81978), PHOX2B (sc376997, Santa Cruz), α-tubulin (T5168, Sigma) and β-actin (ab6276, Abcam).

Secondary antibodies used for chemiluminescent detection were donkey anti-rabbit-HRP (GE healthcare, NA 9340 V), donkey anti-sheep/goat-HRP (Bio-Rad, STAR88P) or sheep anti-mouse-HRP (GE Healthcare, NXA931). Chemiluminescent detection was done using the ECL Prime Western Blotting kit (GE Healthcare, RPN2232) and developed on a ImageQuant LAS 4000 (GE Healthcare, 28–9558–10). For infrared fluorescent detection, membranes were incubated with secondary antibodies donkey anti-rabbit-IRDye® 800CW (Rockland, 611–731–127) or Goat anti-mouse-IRDye® 680RD (LI-COR, 926–68070) and scanned on an Odyssey Infrared imaging System, (LI-COR, LIC-9201–00).

### Transwell migration assay

Cells were resuspended in serum free DMEM and seeded in ThinCert 24 well transwells (8 μm pore size, Greiner). DMEM with 10% FCS was added as chemo-attractant for migrating cells. Non-migrated cells were removed with a cotton swab. Migrated cells were washed with PBS, pre-fixed for 10 min in paraformaldehyde, then fixed in steps of 50 and 100% methanol and stained with 0.1% crystal violet. The number of migrated cells per high-power microscopic field was determined. For each experimental condition, a minimum number of *n* = 3 transwells were analyzed and 3–4 high-power microscopic fields per transwell were quantified. Two-sided Student’s *t*-test assuming equal variance was used to calculate statistical difference.

### In vivo tumorigenicity and histological analysis

For tumor growth assays, 2.5 × 10^6^ SH-SY5Y-TetR-NOTCH3-IC cells were suspended in 200 μl of a 50% Matrigel (BD, 354234) solution in PBS and subcutaneously injected in female NU/NU nude mice (Crl:NU-Foxn1nu, 6–8 weeks old, 20–30 g) (Charles Rivers). After tumor outgrowth to around 200–250 mm^3^, mice were randomized to control or treatment groups. Doxycycline was added to the drinking water at a final concentration of 200 µg/ml to induce expression of NOTCH3-IC. Tumor size was measured using a caliper. Two-sided Student’s *t*-test assuming equal variance was used to calculate statistical difference in size of control and doxycycline-treated tumors. Mice were sacrificed at day 7 or day 14 after start of doxycycline treatment, the tumors were isolated and divided in two parts. One tumor piece was fresh frozen in liquid nitrogen and RNA was isolated using Trizol reagent. The other tumor piece was fixed in 4% (w/v) buffered formaldehyde (Klinipath) and embedded in paraffin for histological analyses. In vivo experiments were conducted after ethical approval from the animal experiments committee of the AMC was obtained.

Paraffin-embedded SH-SY5Y-TetR-NOTCH3-IC neuroblastoma tumors where sectioned at 4 μm and processed by standard immunohistochemistry protocols. For NCAM1 (CD56) and MAML2 staining, antigen retrieval was performed with 10 mM Tris- 1 mM EDTA pH 9.0 and sections were incubated overnight at 4 °C with rabbit monoclonal NCAM1 (CD56) antibody (MRQ-42, Cell Marque, 1:100 dilution) or rabbit polyclonal MAML2 antibody (#4618, Cell Signaling Technology, 1:100). This was followed by incubation with secondary Brightvision poly-anti-Rabbit IgG antibody (Immunologic). Stainings were developed using Bright DAB+ detection kit (Immunologic) and slides were counterstained with 1:10 Hematoxylin (Klinipath).

### Reporting summary

Further information on experimental design is available in the Nature Research Reporting Summary linked to this article.

## Supplementary information

Supplementary Information

Description of Additional Supplementary Files

Supplementary Data 1

Supplementary Data 2

Reporting Summary

Source Data

## Data Availability

The Affymetrix gene expression data and ChIP-sequencing data generated in this study are available from GEO (accession number GSE116893). Previously generated mRNA expression profiles of MES and ADRN cells are available from GEO (accession number GSE90805^1^). RNA-sequencing data from 498 primary neuroblastoma^22,23^ is available from GEO (accession number GSE62564). The source data underlying Figs. 1c, 2a, b, 3c, 5b, c, 6a and 7a are provided as a Source Data file. Requests for material can be send to J.v.N.
