# Supplementary material for: A NOTCH feed-forward loop drives reprogramming from adrenergic to mesenchymal state in neuroblastoma
Source: Nat Commun. 2019 Apr 4;10:1530. doi: 10.1038/s41467-019-09470-w (PMC6449373; doi:10.1038/s41467-019-09470-w)
Supplement: Supplementary file 1 — Supplementary Information [file 41467_2019_9470_MOESM1_ESM.pdf]

Supplementary Information

A NOTCH feed-forward loop drives reprogramming from adrenergic- to mesenchymal state in neuroblastoma

van Groningen et al.

Supplementary Figure 1

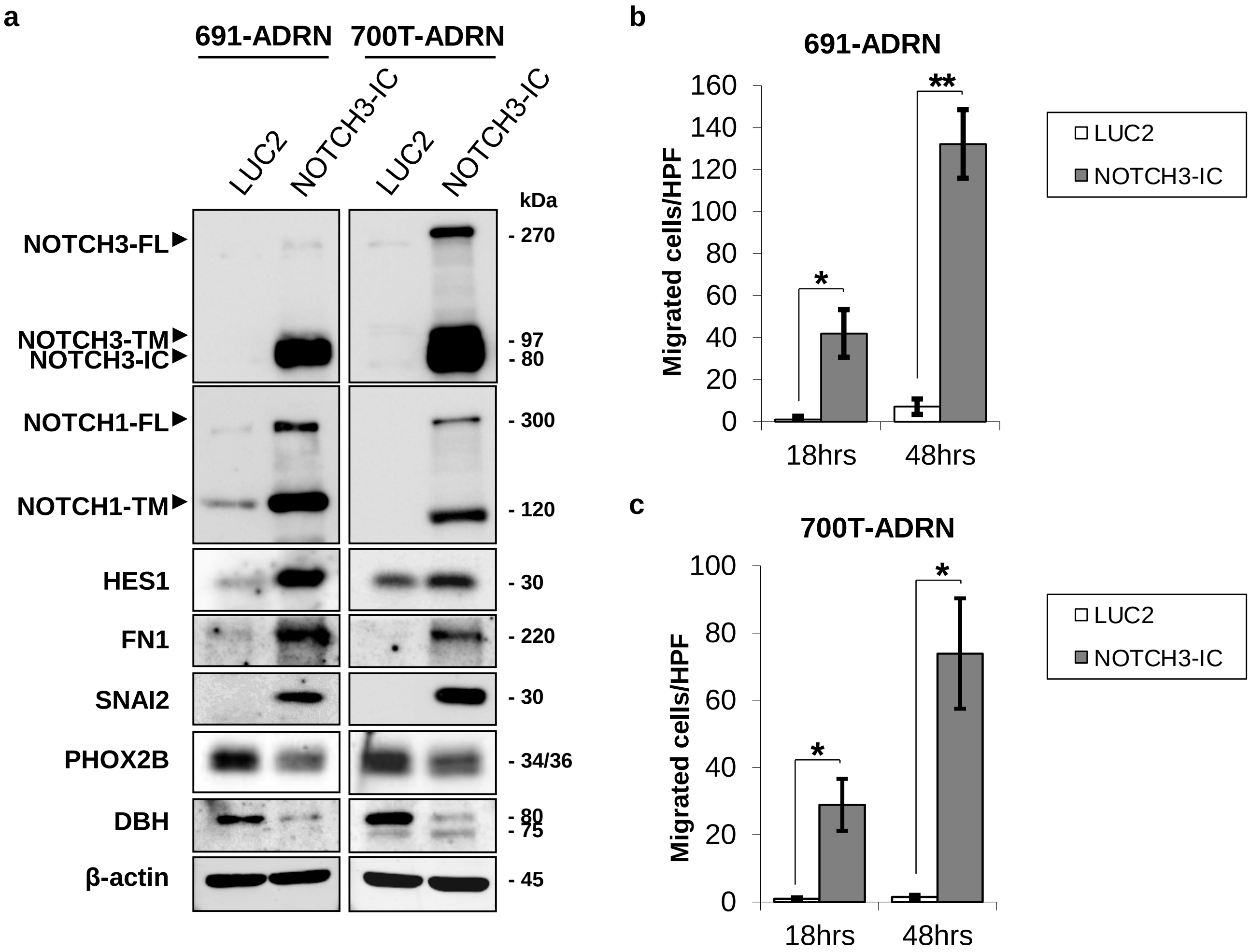

NOTCH3-IC induces a MES phenotype in cell lines with MYCN amplification

a. Western blot analysis of 691-ADRN (left) and 700T-ADRN (right) transduced with NOTCH3-IC or a luciferase2 (LUC2) control cDNA for T=96 hours. Analyzed markers include NOTCH pathway genes (NOTCH3, NOTCH1 and HES1), MES-markers (FN1, SNAI2) and ADRN-markers (PHOX2B, DBH). β-actin was used as loading control. Note that both 691-ADRN and 700T-ADRN have amplification of the MYCN oncogene. **b, c.** Transwell migration assay of **b)** 691-ADRN and **c)** 700T-ADRN transduced with NOTCH3-IC (grey bars) or a luciferase2 (LUC2) control cDNA (white bars). Cells were allowed to migrate for 18 or 48 hours after seeding in transwells. Error bars represent standard deviation. Two-sided Student's *t*-test assuming equal variance was used to calculate statistical significance, * $p \leq 0.05$, ** $p \leq 0.01$.

Supplementary Figure 2

a

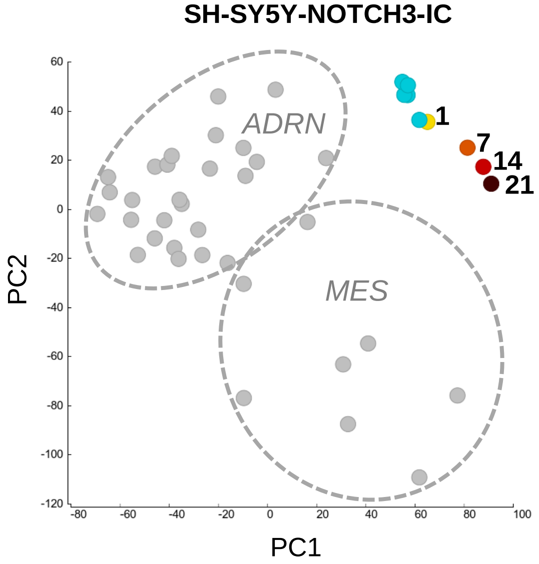

b

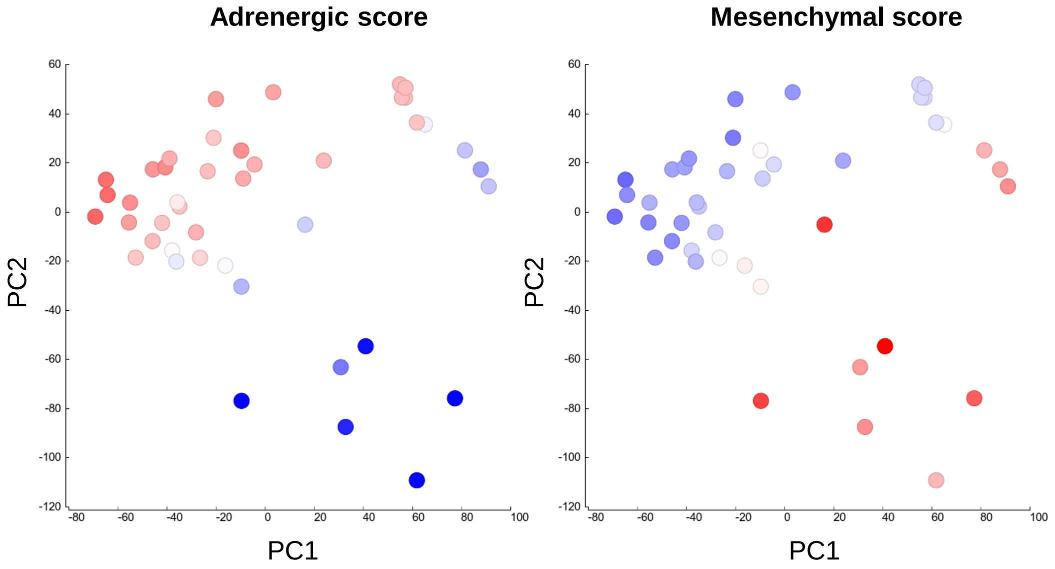

Principal Component Analysis of neuroblastoma cell lines and SH-SY5Y cells with inducible NOTCH3-IC expression

a. Principal Component Analysis (PCA) of mRNA expression profiles from neuroblastoma cell lines and SH-SY5Y cells with inducible NOTCH3-IC expression. PC1 and PC2 indicate principal components 1 and 2, respectively. Dashed lines group cell lines with adrenergic (ADRN) or mesenchymal (MES) phenotype according to reference[1]. Non-induced control SH-SY5Y-NOTCH3-IC cells are shown in blue. Cells with 1, 7, 14 or 21 days of NOTCH3-IC induction are shown in yellow, orange, red and brown, respectively. **b.** Superimposed signature scores for adrenergic and mesenchymal cells plotted on the PCA of cell lines shown in **a**.

Supplementary Figure 3

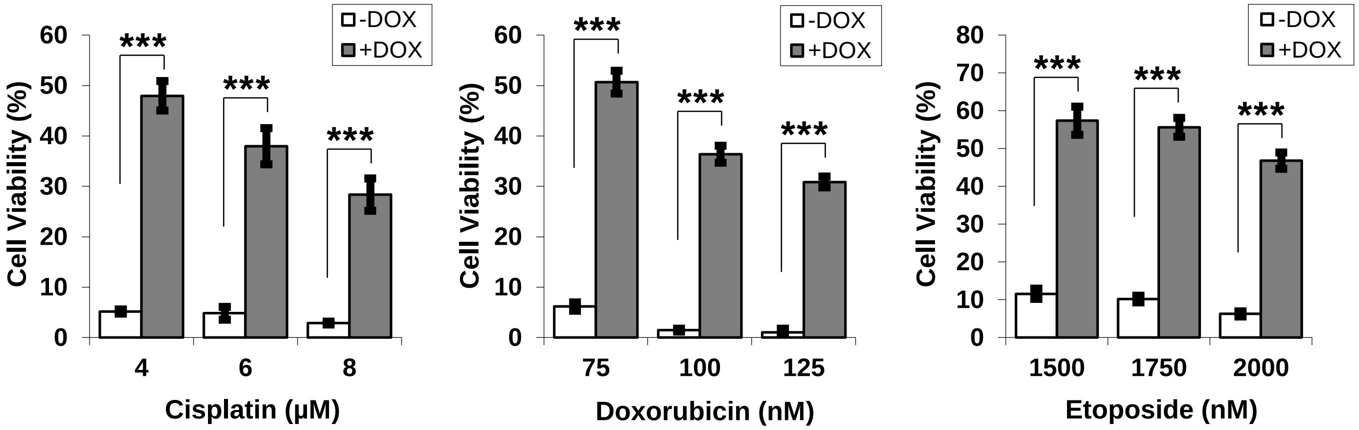

NOTCH3-IC induces resistance to chemotherapy

Bar plots showing sensitivity to cisplatin, doxorubicin or etoposide in SH-SY5Y with (+dox) or without (-dox) induction of NOTCH3-IC. MTT-assays were used to measure cell viability after treatment with increasing concentrations of each chemotherapeutic drug. dox, doxycycline. Error bars represent standard deviation. Two-sided Student's *t*-test assuming equal variance was used to calculate statistical significance, *** $p \leq 0.001$.

Supplementary Figure 4

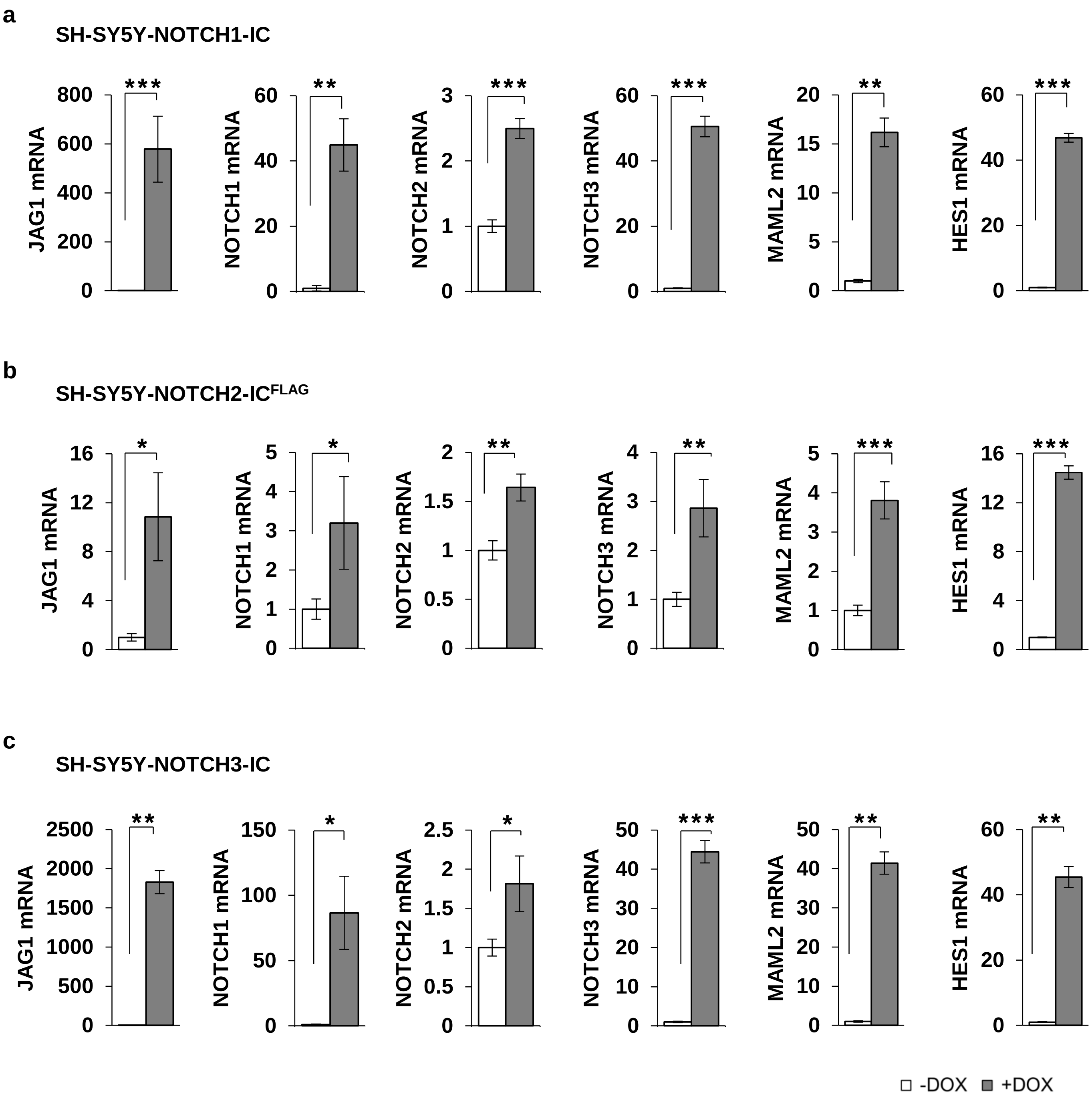

A transcriptional feed-forward cascade in the NOTCH pathway

a-c. qRT-PCR analysis on SH-SY5Y cells with inducible expression of **(a)** NOTCH1-IC, **(b)** NOTCH2-IC^{FLAG} or **(c)** NOTCH3-IC for T=7 days. Each cell line was analyzed for expression of JAG1, NOTCH1, NOTCH2, NOTCH3, MAML2 and HES1. Shown is relative mRNA expression compared to non-induced control cells (-dox, white bars). Expression in cells with NOTCH-IC induction (+dox) is depicted in grey bars. Error bars represent standard deviation. Two-sided Student's *t*-test assuming equal variance was used to calculate statistical significance, * $p \leq 0.05$, ** $p \leq 0.01$, *** $p \leq 0.001$.

Supplementary Figure 5

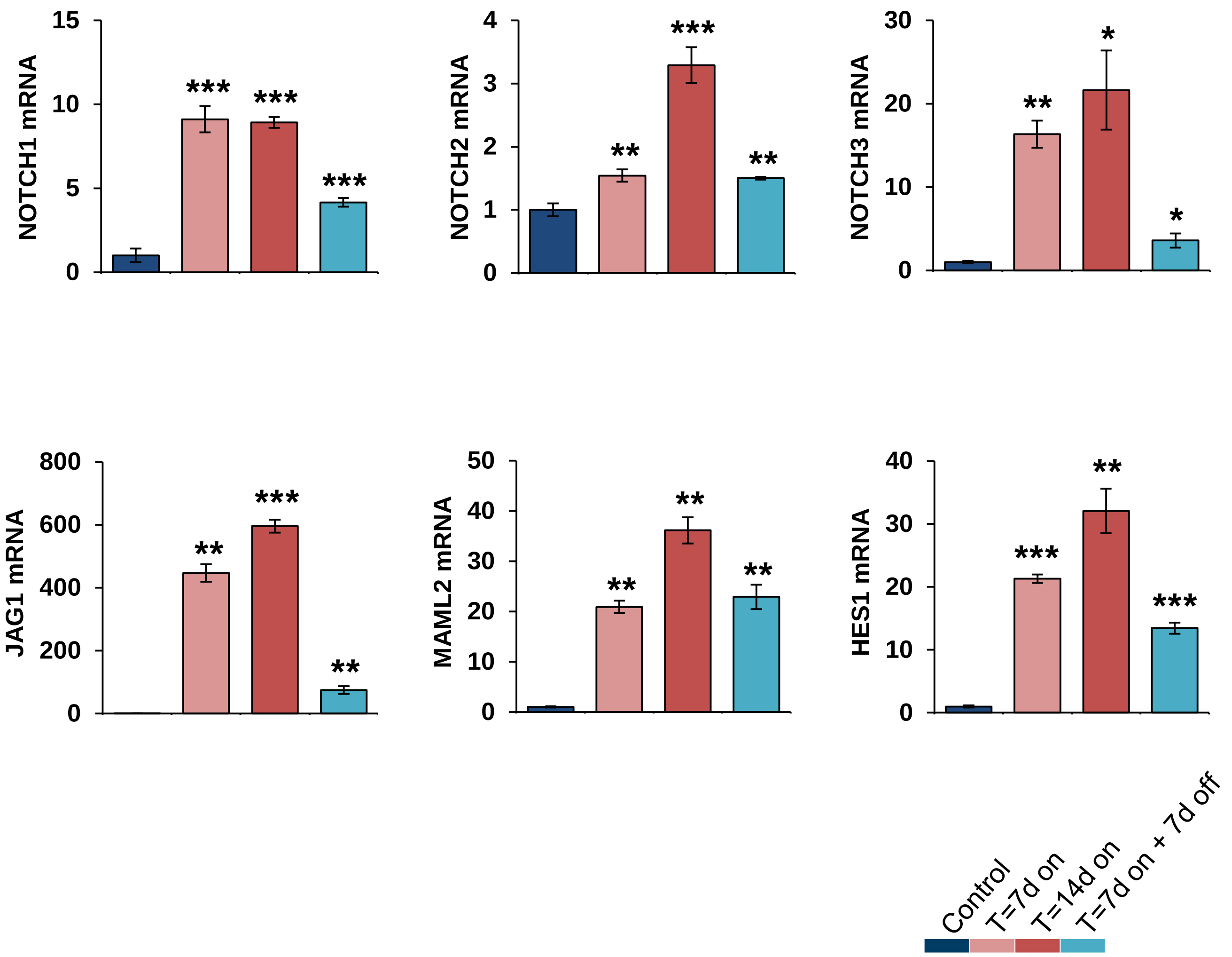

Transient NOTCH3-IC expression induces a NOTCH feed-forward cascade

qRT-PCR analysis of NOTCH1, NOTCH2, NOTCH3, JAG1, MAML2 and HES1 in SH-SY5Y-NOTCH3-IC cells. Data show relative mRNA expression as compared to non-induced control cells (-dox), depicted in dark blue. Samples harvested after a continued NOTCH3-IC induction of 7 or 14 days are shown in pink and red, respectively. RNA harvested after a transient phase of T=7 days NOTCH3-IC induction and followed by T=7 days doxycycline wash-out from the culture medium, are shown in light blue. Error bars represent standard deviation. Two-sided Student's *t*-test assuming equal variance was used to calculate statistical significance, * $p \leq 0.05$, ** $p \leq 0.01$, *** $p \leq 0.001$. The significance of regulation is relative to non-induced control cells.

Supplementary Figure 6

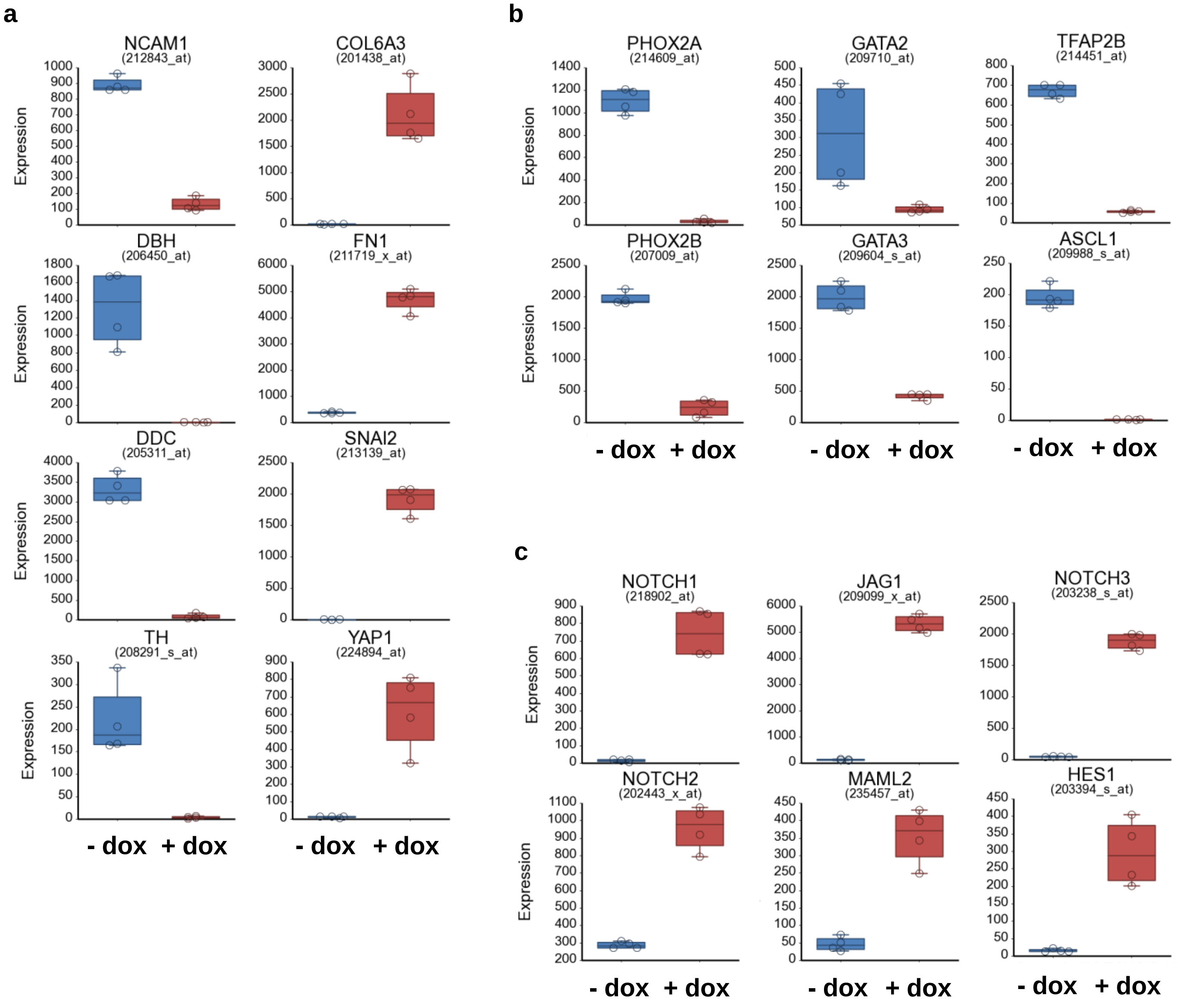

Lineage reprogramming *in vivo*

a-c. mRNA expression analysis in SH-SY5Y-NOTCH3-IC xenograft tumors with NOTCH3-IC induction (+dox, in red) as compared to non-induced control tumors (-dox, in blue). Two pairs of –dox and +dox tumors were analyzed at T=7 days of induction and two of such pairs were analyzed at T=14 days of induction. Expression data of tumors with T=7 and T=14 days induction reveal consistent patterns of mRNA regulation and are shown in a single dot-boxplot for clarity. Whiskers denote the interval within 1.5 times the interquartile range (box edges) of the median (center line). Each tumor is represented by an open circle. Shown is expression of **a)** ADRN-markers (NCAM1, DBH, DDC, TH) and MES-markers (COL6A3, FN1, SNAI2, YAP1), **b)** core transcription factors of ADRN-type cells and **c)** genes from the NOTCH signaling route. dox, doxycycline. mRNA was measured by Affymetrix gene expression arrays.

Supplementary Figure 7

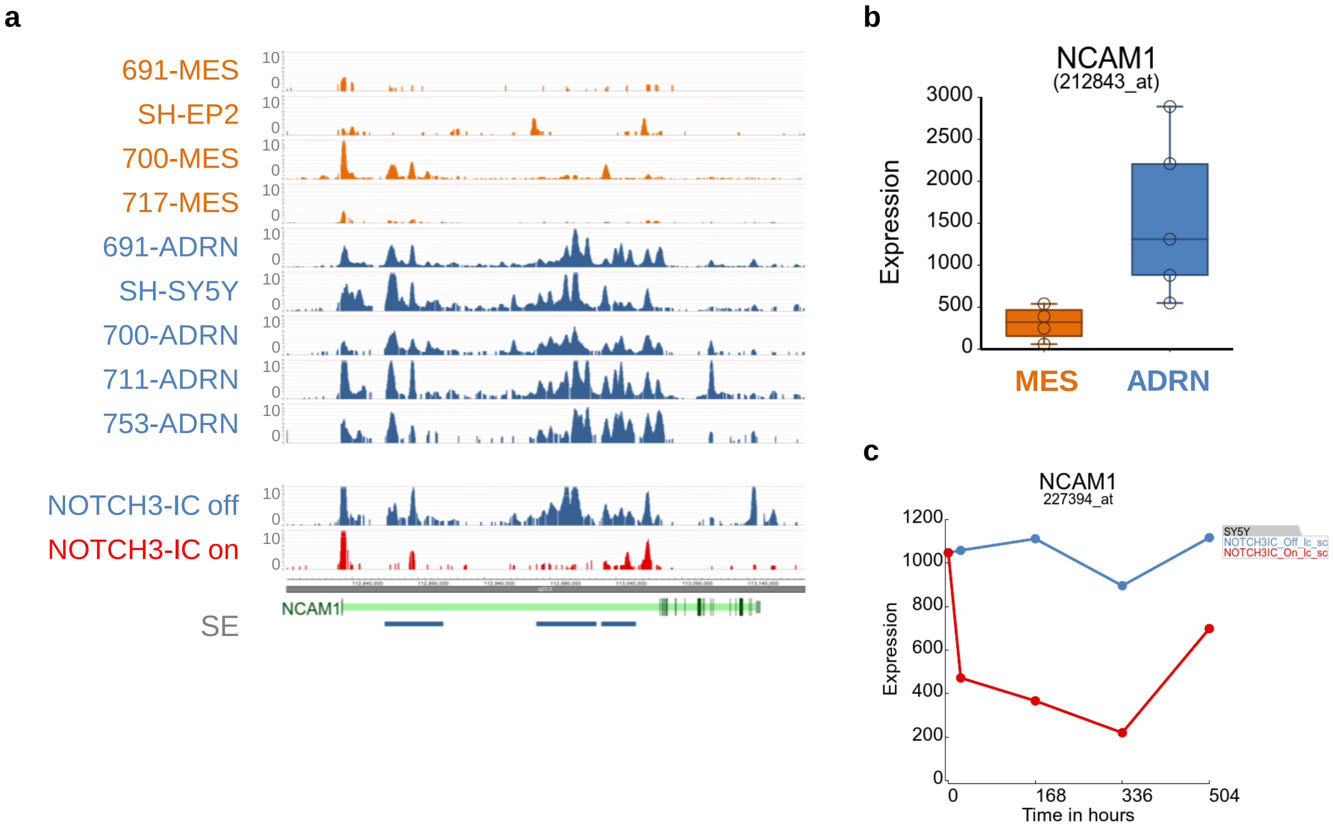

Identification of NCAM1 as ADRN marker for ADRN-to-MES reprogramming

a. ChIP-sequencing profiles of H3K27ac in cell lines of MES-type (691-MES, SH-EP2, 700-MES, 717-MES, in orange), ADRN-type (691-ADRN, SH-SY5Y, 700-ADRN, 711-ADRN, 753-ADRN, in blue), or SH-SY5Y cells with inducible NOTCH3-IC expression (-dox control in blue, +dox in red; T=7days of induction). The number of reads per 20 million mapped reads is shown on the y-axis. Chromosomal gene position is shown on the x-axis. ADRN-specific super-enhancers (SE) are indicated by blue horizontal bars. **b.** Box plot of NCAM1 mRNA expression for the cell lines from panel A that are grouped in four MES cell lines (orange) and five ADRN cell lines (blue). Whiskers denote the interval within 1.5 times the interquartile range (box edges) of the median (center line). Open circles indicate expression values for each individual cell line. Affymetrix mRNA probeset of NCAM1 is indicated. **c.** Time course mRNA analysis of NCAM1 expression in SH-SY5Y-NOTCH3-IC cells with (+ dox, in red) induction of NOTCH3-IC. Non-induced control cells (- dox) are depicted in blue. Each measurement is represented by a dot and dots are connected with a line. Time (in hours) is depicted on the x-axis; mRNA expression values are depicted on the y-axis.

Supplementary Figure 8

a

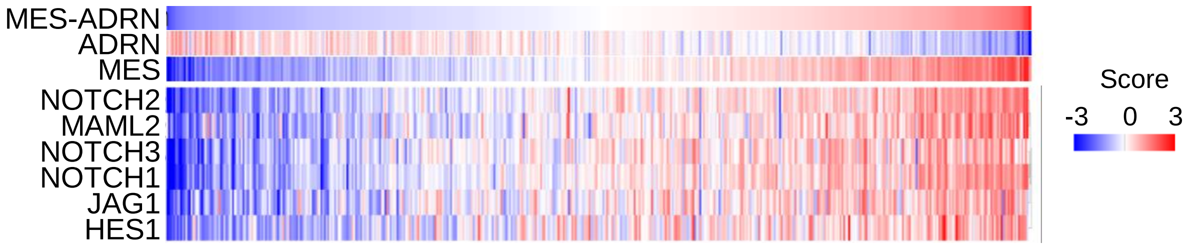

b

Gene	R^2 with MES signature	*P*-value
NOTCH1	0.800	6.6×10^{-112}
NOTCH2	0.825	3.7×10^{-125}
NOTCH3	0.725	2.9×10^{-82}
JAG1	0.681	4.8×10^{-69}
MAML2	0.747	4.4×10^{-90}
HES1	0.668	1.1×10^{-65}

c

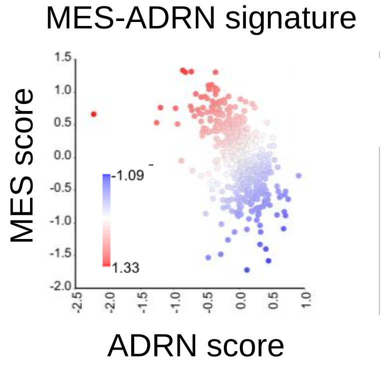

d

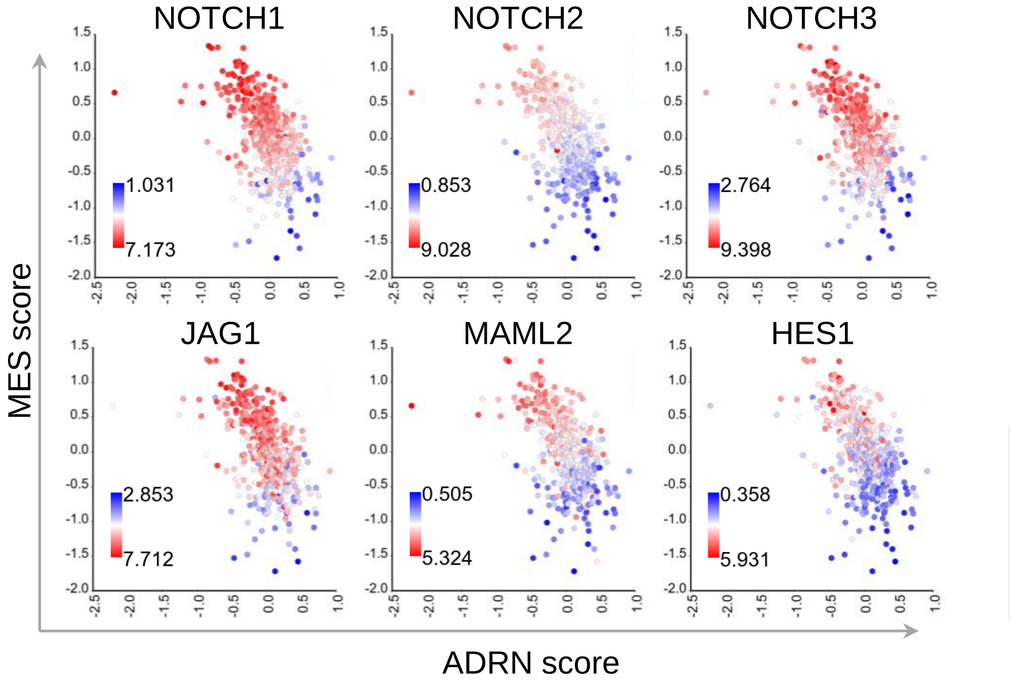

Expression of NOTCH pathway genes correlates with a MES expression state in human neuroblastoma

a. Heatmap showing scores of MES and ADRN gene expression signatures in a series of 498 human neuroblastoma. Samples are ordered by increasing values of a weighted-matched and merged MES-ADRN signature score. In the same order, the normalized gene expression values of NOTCH1, NOTCH2, NOTCH3, JAG1, MAML2 and HES1 are shown for each individual tumor. **b.** Correlations of the MES signature with expression of genes of the NOTCH feed-forward cascade (NOTCH1, NOTCH2, NOTCH3, JAG1, MAML2 and HES1) in 498 neuroblastoma. Significance of correlation was determined by 2-way ANOVA. **c, d.** Visualization of **c)** the weighted-matched MES-ADRN signature score and **d)** 2log-transformed expression of NOTCH1, NOTCH2, NOTCH3, JAG1, MAML2 and HES1 on 498 human neuroblastoma. Each tumor is represented by a dot that is plotted on a 2-dimensional space using ADRN and MES signature scores on the x- and y-axis, respectively.

Supplementary Reference

1. van Groningen, T. *et al.* Neuroblastoma is composed of two super-enhancer-associated differentiation states. *Nat Genet* **49**, 1261-1266 (2017).
