# Supplementary material for: A NOTCH feed-forward loop drives reprogramming from adrenergic to mesenchymal state in neuroblastoma
Source: Nat Commun. 2019 Apr 4;10:1530. doi: 10.1038/s41467-019-09470-w (PMC6449373; doi:10.1038/s41467-019-09470-w)
Supplement: Supplementary file 2 — Description of Additional Supplementary Files [file 41467_2019_9470_MOESM2_ESM.pdf]

Description of Additional Supplementary Information

File Name: Supplementary Data 1

Description: Regulation of MES-ADRN signature genes by NOTCH3-IC

File Name: Supplementary Data 2

Description: Motif analysis on increased H3K27ac regions after NOTCH3-IC induction
