# Supplementary material for: A NOTCH feed-forward loop drives reprogramming from adrenergic to mesenchymal state in neuroblastoma
Source: Nat Commun. 2019 Apr 4;10:1530. doi: 10.1038/s41467-019-09470-w (PMC6449373; doi:10.1038/s41467-019-09470-w)
Supplement: Supplementary file 5 — Reporting Summary [file 41467_2019_9470_MOESM5_ESM.pdf]

nature research

Corresponding author(s): *Double-blind peer review submissions: write DBPR and your manuscript number here instead of author names.*

Reporting Summary

Nature Research wishes to improve the reproducibility of the work that we publish. This form provides structure for consistency and transparency in reporting. For further information on Nature Research policies, see Authors & Referees and the Editorial Policy Checklist.

Statistical parameters

When statistical analyses are reported, confirm that the following items are present in the relevant location (e.g. figure legend, table legend, main text, or Methods section).

n/a	Confirmed	
☐	☒	The exact sample size (n) for each experimental group/condition, given as a discrete number and unit of measurement
☐	☒	An indication of whether measurements were taken from distinct samples or whether the same sample was measured repeatedly
☐	☒	The statistical test(s) used AND whether they are one- or two-sided<br>*Only common tests should be described solely by name; describe more complex techniques in the Methods section.*
☐	☒	A description of all covariates tested
☐	☒	A description of any assumptions or corrections, such as tests of normality and adjustment for multiple comparisons
☐	☒	A full description of the statistics including central tendency (e.g. means) or other basic estimates (e.g. regression coefficient) AND variation (e.g. standard deviation) or associated estimates of uncertainty (e.g. confidence intervals)
☐	☒	For null hypothesis testing, the test statistic (e.g. F, t, r) with confidence intervals, effect sizes, degrees of freedom and P value noted<br>*Give P values as exact values whenever suitable.*
☒	☐	For Bayesian analysis, information on the choice of priors and Markov chain Monte Carlo settings
☒	☐	For hierarchical and complex designs, identification of the appropriate level for tests and full reporting of outcomes
☒	☐	Estimates of effect sizes (e.g. Cohen's d, Pearson's r), indicating how they were calculated
☐	☒	Clearly defined error bars<br>*State explicitly what error bars represent (e.g. SD, SE, CI)*

Our web collection on statistics for biologists may be useful.

Software and code

Policy information about availability of computer code

Data collection	RSEG and ROSE algorithms are publicly available
Data analysis	RSEG and ROSE algorithms are publicly available

For manuscripts utilizing custom algorithms or software that are central to the research but not yet described in published literature, software must be made available to editors/reviewers upon request. We strongly encourage code deposition in a community repository (e.g. GitHub). See the Nature Research guidelines for submitting code & software for further information.

Data

Policy information about availability of data

All manuscripts must include a data availability statement. This statement should provide the following information, where applicable:
- Accession codes, unique identifiers, or web links for publicly available datasets
- A list of figures that have associated raw data
- A description of any restrictions on data availability

Data is available from GEO, accession codes GSE116893 and GSE90805, accession codes are included in methods and data avilability sections

Field-specific reporting

Please select the best fit for your research. If you are not sure, read the appropriate sections before making your selection.

☒ Life sciences ☐ Behavioural & social sciences ☐ Ecological, evolutionary & environmental sciences

For a reference copy of the document with all sections, see nature.com/authors/policies/ReportingSummary-flat.pdf

Life sciences study design

All studies must disclose on these points even when the disclosure is negative.

Sample size	A minimum number of 4 samples was required in each group to perform two-sided t tests. Cell growth and resistance experiments were repeated at least twice. Sample size was chosen to reach a minimum power of 80%.
Data exclusions	No data was excluded for analysis
Replication	Growth and resistance assays were repeated at least twice. Gene expression profiling of reprogramming by NOTCH3-IC was repeated twice.
Randomization	N/A
Blinding	Histological analyses were blinded to investigator.

Reporting for specific materials, systems and methods

Materials & experimental systems

n/a	Involved in the study
☐	☒ Unique biological materials
☐	☒ Antibodies
☐	☒ Eukaryotic cell lines
☒	☐ Palaeontology
☐	☒ Animals and other organisms
☒	☐ Human research participants

Methods

n/a	Involved in the study
☐	☒ ChIP-seq
☒	☐ Flow cytometry
☒	☐ MRI-based neuroimaging

Unique biological materials

Policy information about availability of materials

Obtaining unique materials	Unique biological materials are available form the corresponding author upon reasonable request

Antibodies

Antibodies used	Chip-seq: H3K27ac (4729, Abcam);<br>Western blot: Primary antibodies NOTCH1 (4380), NOTCH1-IC (4147), NOTCH2 (4530), NOTCH3 (5276), MAML2 (6988), HES1 (11988), DBH (8586), VIM (5741), SLUG, (9585), JAGGED1 (2620), GATA2 (4595), YAP1 (4912), SOX9 (82630), TFAP2B (2509), PARP (9542) and FLAG (2368) were obtained from Cell Signaling Technology. Other primary antibodies were FN1 (AF1918, R&D systems), PHOX2A (sc81978), PHOX2B (sc376997, Santa Cruz), α-tubulin (T5168, Sigma) and β-actin (ab6276, Abcam).<br>Secondary antibodies used for chemiluminescent detection were donkey anti-rabbit-HRP (GE healthcare, NA 9340V), donkey anti-sheep/goat-HRP (Bio-Rad, STAR88P) or sheep anti-mouse-HRP (GE Healthcare, NXA931).<br>For infrared fluorescent detection, membranes were incubated with secondary antibodies donkey anti-rabbit-IRDye® 800CW (Rockland, 611-731-127) or Goat anti-mouse-IRDye® 680RD (LI-COR, 926-68070).<br>Immunohistochemistry: NCAM1 (CD56) antibody (MRQ-42, Cell Marque, 1:100 dilution) or rabbit polyclonal MAML2 antibody (#4618, Cell Signaling Technology, 1:100).
Validation	Antibodies have been validated by manufacturers and in our previous study (van Groningen et al., Nat Gen 2017).

Eukaryotic cell lines

Policy information about cell lines

Cell line source(s)	Cell lines were derived from neuroblastoma patients (see van Groningen et al., 2017 and references therein)
Authentication	Cell line authenticity was verified using Short Tandem Repeat (STR) analysis.
Mycoplasma contamination	Cells were routinely checked for the presence of mycoplasma using MycoAlert detection kit (Lonza).
Commonly misidentified lines (See ICLAC register)	N/A

Animals and other organisms

Policy information about studies involving animals; ARRIVE guidelines recommended for reporting animal research

Laboratory animals	Female NU/NU nude mice (Crl:NU-Foxn1nu, 6–8 weeks old, 20–30 g) (Charles Rivers)
Wild animals	N/A
Field-collected samples	N/A

ChIP-seq

Data deposition

☒ Confirm that both raw and final processed data have been deposited in a public database such as GEO.

☒ Confirm that you have deposited or provided access to graph files (e.g. BED files) for the called peaks.

Data access links *May remain private before publication.*	Data is available from GEO, accession codes GSE116893 and GSE90805
Files in database submission	See description in GEO datasets GSE116893 and GSE90805
Genome browser session (e.g. UCSC)	Bio-informatic analysis of microarray data was conducted using R2 (http://R2.amc.nl).

Methodology

Replicates	We perfomed a single H3K27ac ChIP-seq experiment that compared cells with inducible expression of NOTCH3-IC with non-induced control cells. Regulated enhancers were compared to enhancers in isogenic MES and ADRN reference cell lines.
Sequencing depth	34-57 million reads were mappable to HG19.
Antibodies	Chip-seq: H3K27ac (4729, Abcam); extensively tested and validated for neuroblastoma in previous work by van Groningen et al., Nat Gen., 2017
Peak calling parameters	RSEG was used for peak calling. ROSE (without stitching) was used on these peaks.
Data quality	N/A on RSEG
Software	RSEG was used for peak calling. ROSE (without stitching) was used on these peaks.
